# Molecular Structural Changes in Alfalfa Detected by ATR-FTIR Spectroscopy in Response to Silencing of *TT8* and *HB12* Genes

**DOI:** 10.3390/ijms19041046

**Published:** 2018-03-31

**Authors:** Yaogeng Lei, Abdelali Hannoufa, David Christensen, Haitao Shi, Luciana L. Prates, Peiqiang Yu

**Affiliations:** 1 Department of Animal and Poultry Science, College of Agriculture and Bioresources, University of Saskatchewan, 51 Campus Drive, Saskatoon, SK S7N5A8, Canada; yal263@usask.ca (Y.L.); david.christensen@usask.ca (D.C.); haitao.shi@usask.ca (H.S.); lul614@mail.usask.ca (L.L.P.); 2 London Research and Development Centre, Agriculture and Argi-Food Canada, 1391 Sandford Street, London, ON N5V 4T3, Canada; Abdelali.Hannoufa@agr.gc.ca

**Keywords:** gene silencing, transgenic alfalfa, ATR-FTR, univariate analysis, multivariate analysis

## Abstract

This study investigated the spectral changes in alfalfa molecular structures induced by silencing of Transparent Testa 8 (*TT8*) and Homeobox 12 (*HB12*) genes with univariate and multivariate analyses. *TT8*-silenced (TT8i), *HB12*-silenced (HB12i) and wild type (WT) alfalfa were grown in a greenhouse under normal conditions and were harvested at early-to-mid vegetative stage. Samples were free-dried and grounded through 0.02 mm sieve for spectra collections with attenuated total reflectance Fourier transform infrared (ATR-FTIR) spectroscopy. Afterwards, both univariate and multivariate analyses were conducted on amide, carbohydrate and lipid regions. Univariate results showed that silencing of TT8 and HB12 genes affected peak heights of most total carbohydrate (TC) and structural carbohydrate (STC), and structural carbohydrate area (STCA) in carbohydrate regions; and β-sheet height, amide areas, and ratios of amide I/II and α-helix/β-sheet in amide region; and symmetric CH2 (SyCH2), asymmetric CH2 (AsCH2) and (a)symmetric CH2 and CH3 area (ASCCA) in the lipid region. Multivariate analysis showed that both hierarchy cluster analysis (HCA) and principal component analysis (PCA) clearly separated WT from transgenic plants in all carbohydrate regions and (a)symmetric CH2 and CH3 (ASCC) lipid region. In the amide region, PCA separated WT, TT8i and HB12i into different groups, while HCA clustered WT into a separate group. In conclusion, silencing of *TT8* and *HB12* affected intrinsic molecular structures of both amide and carbohydrate profiles in alfalfa, and multivariate analyses successfully distinguished gene-silenced alfalfa from its parental WT control.

## 1. Introduction

Fourier transform infrared (FTIR) spectroscopy is an analytical technique that uses a polychromatic light source allowing for simultaneous collection of spectral absorption data from a wide range of electromagnetic spectra [1]. The absorption data is closely correlated to the vibrational intensities of the molecular bonds of chemical functional groups of samples [2]. Compared with conventional wet analysis, FTIR is rapid, non-destructive and requires no chemical production and no particular preparations [3]. The FTIR has been widely accepted and used in many fields, such as biodiesel [4], food science [5], medical research [6], and plant science [7]. Moreover, FTIR spectroscopy requires only small little amounts of samples [8], which is very useful for the preliminary evaluation of forage quality at the early stages of genetic breeding. FTIR spectroscopy can be divided into three categories according to its spectroscopic sampling mode; transmission, transflection and attenuated total reflection (ATR) [9]. In ATR-FTIR spectroscopy, the FTIR beam goes through a crystal and reflects at the interface of the crystal and the sample on it. The reflection of IR beam creates an evanescent wave (4~6 µm) which can penetrate the sample on the crystal surface [7,9]. Recently, this technique has been used to detect the molecular changes induced by genetic modifications. Heendeniya and Yu [10] applied this technique to dual-transgenic (*Lc* and *C1*) alfalfa (*Medicago sativa*) and found that transgenic alfalfa had higher amide area and amide I/II height ratios, and lower heights in some carbohydrate peaks. Secondary protein structures were also analyzed in this project, and α-helix/β-sheet height ratio was found higher in dual-transgenic alfalfa.

Alfalfa (*Medicago sativa*), also called Lucerne, is one of the most cultivated legume forages worldwide. Due to its high nutritive values and good palatability, it is known as the “queen of fodder” and is wildly fed to high production dairy cows [11]. However, alfalfa contains relatively high lignin content that is barely degradable in the rumen and also could hinder the degradation of other compounds [1]. Efforts has been made in reducing the lignin content in alfalfa [12,13,14], with downregulating the expression levels of genes involving the lignin biosynthesis. *TT8* (*Transparent Testa8*) and *HB12* (*Homeobox 12, Homeodomain Leucine Zipper Class I*) are two transcriptional factors in the phenylpropanoid pathway, which serves as the source of lignin and many secondary metabolites. Observations obtained from *Brassica napus* showed a positive relationship between the expression level of *TT8* and *HB12* [15]. Therefore, our group generated two genotypes of transgenic alfalfa, TT8i and HB12i, with silenced *TT8* and *HB12* genes, respectively. A pilot study previously reported on the carbohydrate structural features and the structural-nutrition relationships in TT8i and HB12i [15,16]. In the current study, we explored univariate structural features in amide, carbohydrate and lipid-related regions. And we also used two multivariate analyses, hierarchical cluster analysis (HCA) and principle component analysis (PCA), on all spectral structural regions in an attempt to distinguish different genotypes.

## 2. Results and Discussion

### 2.1. Carbohydrate Structure-Related Spectral Profiles

The carbohydrate structural parameters of transgenic and wild type (WT) alfalfa are shown in Table 1. Three of the four major total carbohydrate (TC) peak heights were affected by alfalfa transformation with TT8 and HB12 RNAi constructs. Although there were no significant differences within transgenic alfalfa genotypes, TC1 peak height was decreased in transgenic alfalfa plants compared to WT (*p* = 0.003). In contrast, both TC2 and TC3 heights were increased compared with WT control (*p* < 0.001). TC1 centers at ca. 1025 cm^−1^, which is related to starch content in samples [17]. Our chemical analysis showed TT8i and HB12i alfalfa plants contained lower starch relative to WT control (data not shown), which was consistent with our spectral results. There were no significant differences between alfalfa plants with regards to cellulosic compounds (CEC) height and area. All structural carbohydrate (STC) peaks, as well as structural carbohydrate area (STCA), were affected by genetic transformation. STC1 peak height was higher in HB12i alfalfa, while TT8i was not significantly different from WT control. HB12i had the highest STC2, STC3, and STC4 peak heights; whereas TT8i was not different from WT in STC2 and STC4. WT alfalfa had the lowest STC3 followed by TT8i. Likewise, STCA was higher in transgenic alfalfa plants with HB12i having the highest value (*p* < 0.001). Chemically, transgenic alfalfa had higher neutral detergent fiber (NDF) and acid detergent fiber (ADF) contents, with HB12i having the highest values (data not shown). Our data suggested a positive correlation exists between the structural heights/areas and the contents of chemical components.

The current results are not in accordance with the results of a previous pilot study by our group, which was conducted on a smaller population size [15]. In that pilot study, either no differences were found in peak heights or differences were opposite to the current study. The discrepancies in the results of the two studies could be attributed to multiple factors. First, the IR spectra were not normalized in the pilot study, which led to low values in peak heights and areas. Variations in sample thickness under ATR-FTIR determination could affect the results, and such variations could be eliminated through the normalization process. Second, only two replicates of each genotype were used in the pilot study. The smaller size of the alfalfa population might lead to sampling error. To test this hypothesis, we intentionally selected two replicates from each alfalfa genotype to redo the univariate analysis (WT, W2 and W3; TT8i, T2 and T3; HB12i, H2 and H3). Results from this intentionally sub-sampling were provide in the supplementary materials (Sup-2, Small population size results). Most spectral parameters, that were found significant differences in the current study, were not significantly different in the re-sampling study. This alteration in the results indicates that the population size did play an important role in the discrepancy between the pilot study and the present study. Notably, an additional peak was found in both TC and STC regions in the current study, which was absent in the pilot study of Li et al. [15,16]. This is because different methods were used to obtaining structural parameters. In the pilot study, only peaks shown in the FTIR spectrum were included in the analysis. However, there was an inconsistency in the wavenumber of the second TC peak, either ca. ~1100 cm^−1^ or ~1075, in published studies [10,15,18,19,20]. This inconsistency indicates that there were individual peaks at these two wavenumbers; however, one of them might have overlapped with other peaks because of the feature of FTIR spectra [21]. Thus, in the present study, second derivatives were used as references to measure the overlapped peaks.

**Figure 1:**
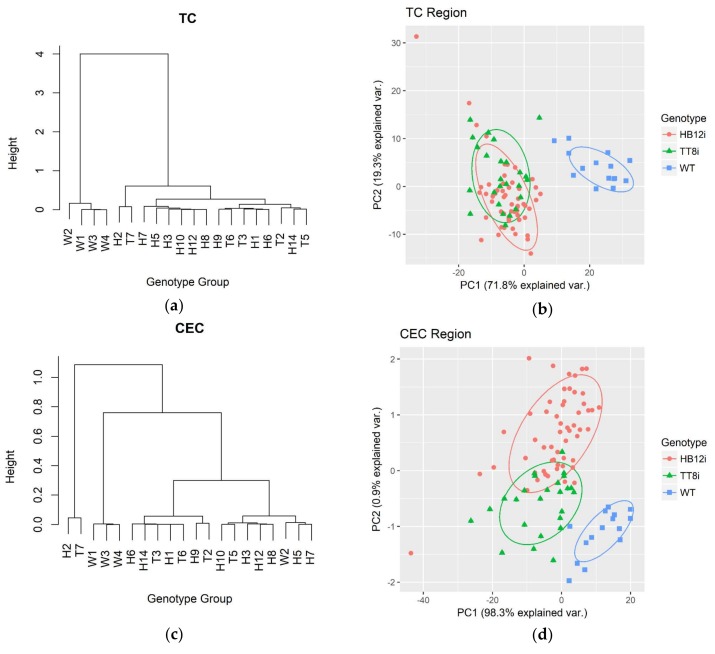

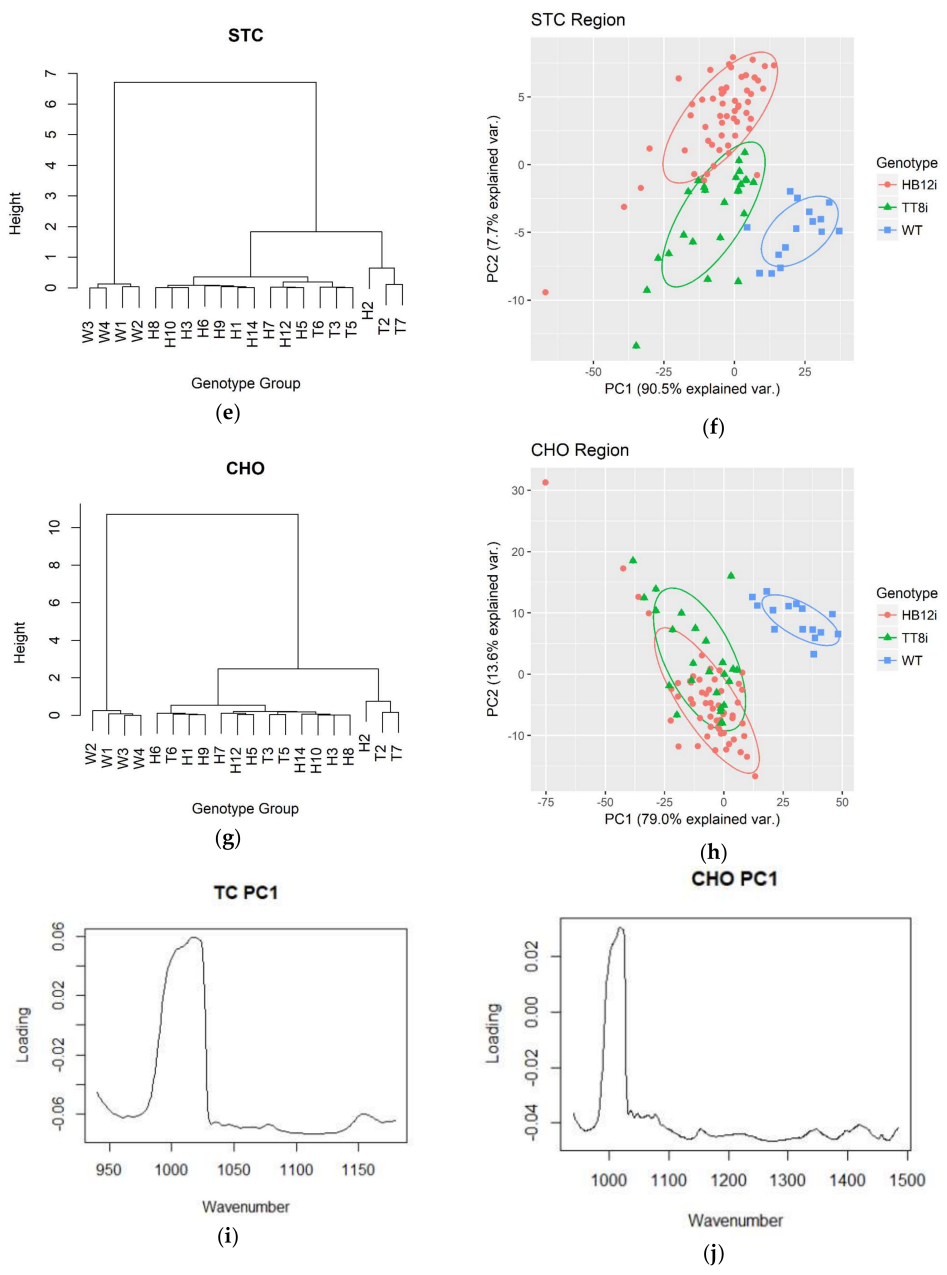
Hierarchy cluster analysis (HCA) dendrograms and principle component analysis (PCA) plots of carbohydrate regions of transgenic (H1–H3, H5–H10, H12, H14; T2–T3, T5–T7) and wild type (WT, W1–W4) control. TC, total carbohydrate (ca. 1178–941 cm^−1^); CEC, cellulosic compounds (ca. 1283–1178 cm^−1^); STC, structural carbohydrate (baseline ca. 1484–1178 cm^−1^); CHO, whole carbohydrate region (ca. 1484–941 cm^−1^). (**a**) HCA dendrogram of TC; (**b**) PCA plot of TC; (**c**) HCA dendrogram of CEC; (**d**) PCA plot of CEC; (**e**) HCA dendrogram of STC; (**f**) PCA plot of STC; (**g**) HCA dendrogram of CHO; (**h**) PCA plot of CHO; (**i**) PC1 loading of TC against wavenumber; (**j**) PC1 loading of CHO against wavenumber.

Plots of HCA and PCA multivariate analyses of three carbohydrate sub-regions (TC, CEC, and TC) and the whole CHO region are shown in Figure 1. Both HCA and PCA clearly separated WT from TT8i and HB12i transgenic plants in all carbohydrate regions. In HCA dendrograms of TC, STC and CHO, WT was clustered in a different group at the heights arounds 4, 7 and 10, respectively. This indicated there were significant differences between WT and transgenic alfalfa in these carbohydrate regions. The HCA dendrogram of CEC region clustered alfalfa populations into three groups at the height of 0.7 with most of WT replicates clustering in a separate group. Nevertheless, HCA clustering failed to separate TT8i and HB12i transgenic alfalfa in carbohydrate regions. Similarly, PCA plots of TC and CHO regions also plotted transgenic alfalfa populations together. However, HB12i and TT8i were distinguished from each other in PCA plots of CEC and STC regions with little overlaps, especially in STC region. Transgenic alfalfa genotypes were separated at the scale of second principle component (PC2). The first principle components of TC, CEC, STC and CHO regions explained 71.8%, 98.3%, 90.5%, 79.0% of population variances, respectively. In our pilot study, Li et al. [16] did multivariate analyses on TC, STC, non-structural carbohydrate (NTC) and CEC regions and found that all genotypes overlapped with each other and were indistinguishable from each other in all carbohydrate regions. This failure in distinguishing alfalfa genotypes could be attributed to the population size and normalization processing.

Multivariate analyses of carbohydrate regions implied that WT differed from transgenic alfalfa populations in every carbohydrate profile. From the PCA plots of TC and CHO region, WT was clearly separated from transgenic alfalfa on PC1 axil with WT at the positive side while transgenic alfalfa at negative side. Thus, we plotted PC1 loading against wavenumber for PCA results of TC and CHO (Figure 1). Except for the region close to ca. 1020 cm^−1^ (around ca. 990–1026 cm^−1^), all other wavenumber variables contributed negatively to PC1.

### 2.2. Amide and Secondary Structure Related Spectral Profiles

Amide region of FTIR spectrum, baseline of ca. 1484–1710 cm^−1^, normally contains two main peaks in high protein samples, amide I and amide II [22]. However, in the current study, amide I and II overlapped and were visibly indistinguishable from each other in most transgenic alfalfa FTIR spectra, which was consistent with Yari et al. [23]. As shown in Table 2, there were no significant differences in amide I peak height between alfalfa populations (*p* = 0.508). In amide II peak height, variance analysis and multiple comparison showed some inconsistency. A significant *p* value of 0.042 was obtained from the F test of variance analysis; however, multiple comparison results among populations were not significant due to the strictness of Tukey-Kramer method. This situation occurs when the *p* value is close to 0.05, and different multiple comparison methods come to different decisions on whether to reject the H0 hypothesis. The amide I to amide II ratio was higher in TT8i (*p* < 0.01), compared to HB12i and WT control. The amide I to amide II ratio in TT8i unveiled the ambiguous results of amide II height, confirming a lower amide II height in TT8i populations.

There were no significant differences in α-helix secondary structures between alfalfa populations. TT8i and HB12i had numerically equal height value of β-sheet, which was higher than that of WT control (*p* < 0.001). The differences of β-sheet carried out to α-helix/β-sheet ratio, as both transgenic alfalfa had lower α-helix/β-sheet ratio with HB12i having the lowest ratio. The higher ratio of β-sheet in transgenic alfalfa could hinder the utilization and availability of protein and reduce protein value. This is because proteins with higher proportion of β-sheet secondary structure are more resistant to enzymatic digestion [24]. Yu et al. [22] evaluated the effects of *Lc* gene transformation, which was aimed at increasing the accumulation of anthocyanidins, on protein secondary structural ratios in alfalfa. Both ratios of α-helix and β-sheet were decreased in *Lc*-transgenic alfalfa.

Transformation with HB12 and TT8 RNAi also affected amide areas (total amide area, AA; amide I area, AIA; and amide II area, AIIA) in alfalfa. HB12i had higher AA compared with TT8i and WT control. Higher AIA and AIIA were also found in HB12i, which were significantly higher than WT and TT8i, respectively. TT8i and WT were not significantly different from each other in terms of AIA and AIIA. The results of our chemical analysis showed lower crude protein (CP) content in both transgenic alfalfa plants with HB12i having the lowest CP, indicating a negative relationship between amide areas and CP content (data not shown). Previous reports in the literature showed both amide heights and areas were positively corelated to CP content in cereals [25,26]. However, Chen et al. [27] reported no significant relationships existed between amide spectral profiles and CP content. There might be more factors affecting the spectral profiles in chemical compositions of feedstuffs, such as sources, types and processing methods of samples. It should be noted that IR spectra in these previous studies were not normalized, which might also contribute to this discrepancy.

**Figure 2:**
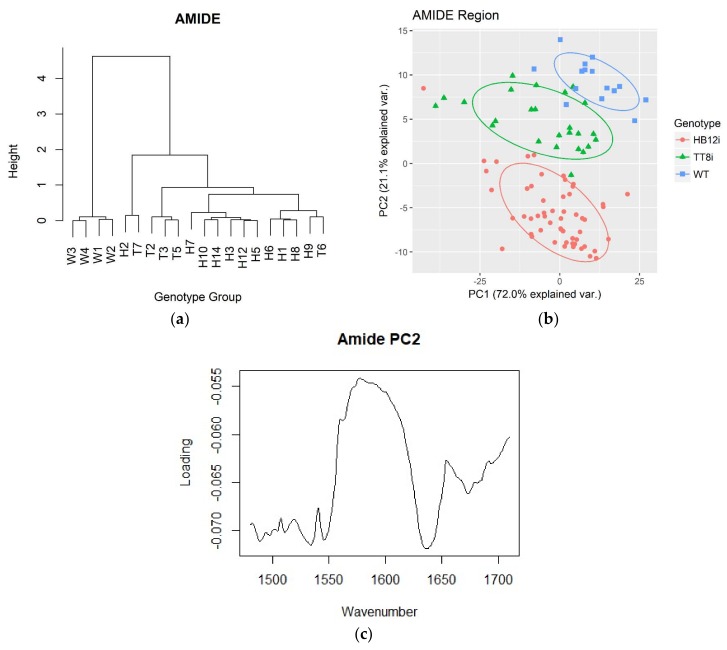
Hierarchy cluster analysis (HCA) dendrogram and principle component analysis (PCA) plot of amide region of transgenic (H1–H3, H5–H10, H12, H14; T2–T3, T5–T7) and wild type (WT, W1–W4) control. Baseline of amide region is ca. 1710–1484 cm^−1^. (**a**) HCA dendrogram of amide region; (**b**) PCA plot of amide region; (**c**) PC2 loading of amide region against wavenumber.

HCA dendrograms and PCA plots of the amide region are shown in Figure 2. In HCA dendrogram, WT was clustered in a group at the height above 4. Moreover, most of HB12i alfalfa sub-genotypes (except for H2) were clustered in a group at the height around 0.8. Similar results were also obtained in PCA plots of the first two PCs, with PC1 and PC2 explained 72.0% and 21.1% of total population variances. WT, HB12i, and TT8i were separated into different ellipses at PC2 axil. Plot of PC2 loadings against wavenumber variables are also shown in Figure 2. All variables negatively contributed to PC2, with variables of ca. 1480–1550 cm^−1^ and regions close to ca.1640 cm^−1^ outweighed others.

### 2.3. Lipid-Related Structure Spectral Profiles

There are two regions relating to lipid profiles of samples in mid infrared (MIR) spectrum, carbonyl C=O ester stretching region (ca. 1710–1781 cm^−1^) and (a)symmetric CH2/3 stretching region (ASCC, ca. 3000–2761 cm^−1^) [28,29,30]. As shown in Table 3, there were no spectral differences in carbonyl C=O ester region, as neither carbonyl C=O (CCO) height nor carbonyl C=O area (CCOA) showed significant differences between alfalfa populations. As to asymmetric and symmetric CH2 and CH3 stretching region (ASCC), HB12i had higher symmetric CH2 (SyCH2) and asymmetric CH2 (AsCH2) heights, compared with TT8i and WT (*p* = 0.004). There were no significant differences were detected in symmetric CH3 (SyCH3) and asymmetric (AsyCH3) peaks among populations. Moreover, HB12i had higher ASCC area (ASCCA) than WT control; in contrast, TT8i was neither different from HB12i nor WT control in ASCCA. Interestingly, similar increases of SyCH2, AsCH2 and ASCCA were found in early-flowering alfalfa compared with early- and late-bud stage [23]. As nutrient values of alfalfa decrease after flowering, these spectral results might indicate lower nutrient availability of HB12i population.

**Figure 3:**
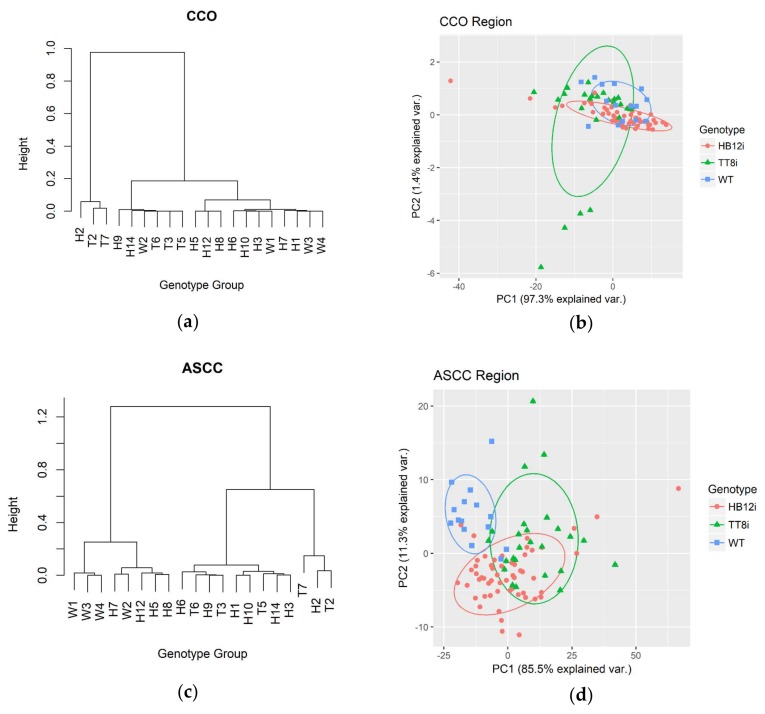
Hierarchy cluster analysis (HCA) dendrograms and principle component analysis (PCA) plots of lipid-related region of transgenic (H1–H3, H5–H10, H12, H14; T2–T3, T5–T7) and WT (W1–W4) control. CCO, carbonyl C=O region (ca. 1710–1781 cm^−1^); ASCC, asymmetric and symmetric CH2 and CH3 region (ca 3000–2761 cm^−1^). (**a**) HCA dendrogram of CCO region; (**b**) PCA plot of CCO region; (**c**) HCA dendrogram of ASCC region; (**d**) PCA plot of ASCC region.

Figure 3 shows HCA dendrograms and PCA plots of lipid-related IR regions. All alfalfa populations were indistinguishable from each other in CCO region by neither HCA nor PCA. However, in ASCC region, WT was separated from its transgenic counterparts in both HCA and PCA. In PCA plots, WT ellipse was only little overlapped with TT8i ellipse and totally separated from HB12i ellipse. PC1 of CCO and AASC regions explained 97.3% and 85.5% of total population variances.

### 2.4. Multivariate Analysis in Fingerprint and Whole Region

**Figure 4:**
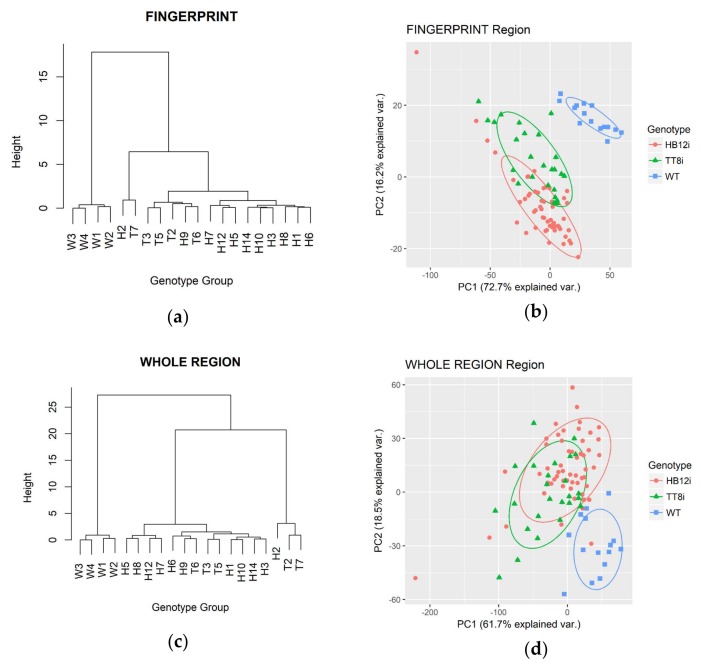
Hierarchy cluster analysis (HCA) dendrograms and principle component analysis (PCA) plots of whole region and fingerprint region of transgenic (H1–H3, H5–H10, H12, H14; T2–T3, T5–T7) and WT (W1–W4) control. Baseline of fingerprint region and whole region are ca. 1800–800 cm^−1^ and ca. 4000–700 cm^−1^, respectively. (**a**) HCA dendrogram of fingerprint region; (**b**) PCA plot of fingerprint region; (**c**) HCA dendrogram of whole FTIR region; (**d**) PCA plot of whole FTIR region.

HCA dendrograms and PCA plots of fingerprint region and whole mid-IR region transgenic and WT control are shown in Figure 4. As shown in the figure, both HCA and PCA clustered WT into a separated group in fingerprint and whole mid-IR regions. In HCA dendrograms, WT was separated from transgenic populations at the heights over 15 and 25 in fingerprint region and whole mid-IR region, respectively. In PCA plots of fingerprint region, PC1 and PC2 explained 72.7% and 16.2% of population variations, respectively. In whole mid-IR region, PCA results showed that PC1 and PC2 explained 61.7% and 18.5% variations, respectively.

## 3. Materials and Methods

### 3.1. RNAi Transformation, Growth Condition and Sampling

Information on the making of RNAi constructs and transformation of alfalfa, growth conditions and sampling methods was previously described in Li et al. [15]. Briefly, total RNA was extracted from alfalfa clone N4.4.2 for cDNA synthesis and assembly of RNAi constructs for TT8 and HB12 using the Gateway and pHellsgate12 vectors. The RNAi constructs were then used to transform alfalfa explants using Agrobacterium tumefaciens according to Aung et al. [31]. The resulting transgenic plants, along with wild type non-transgenic control (WT), were initially grown in a growth chamber and then transplanted to the greenhouse under 21–23 ℃, 16/8 h light/dark rhythm, and 70% of humidity. Alfalfa plants were harvested at early-to-mid vegetative stage about 3 cm above the ground. Alfalfa plant samples were freeze-dried in plastic bags with each bag containing plants from one growing pot. There were 5 bags of TT8 RNAi (TT8i, *n* = 5), 11 bags of HB12 RNAi (HB12i, *n* = 11) and 4 bags of WT (*n* = 4). Each bag of each genotype was considered as one biological replicate for the genotype.

### 3.2. ATR-FTIR Spectroscopy

**Figure 5:**
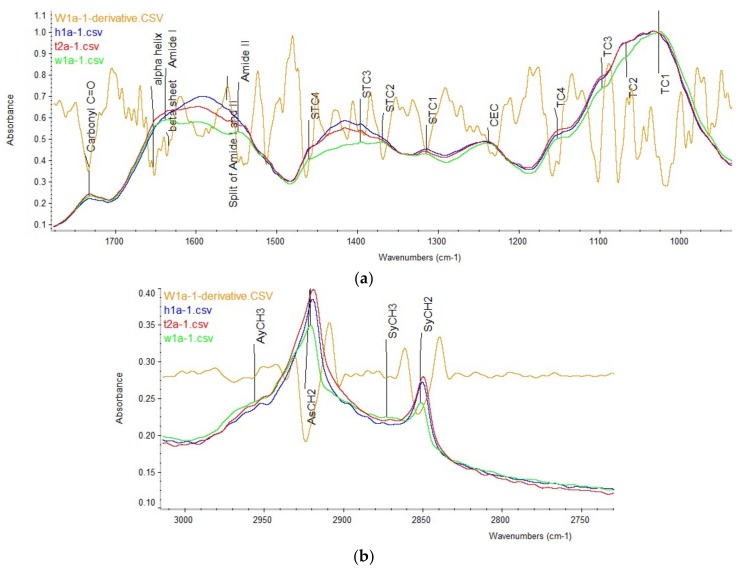
Spectra of transgenic (H1–H3, H5–H10, H12, H14; T2–T3, T5–T7) and WT (W1–W4) control with annotations. (**a**) Carbonyl C=O (CCO), Amide and carbohydrate (CHO) region; (**b**) Symmetric and asymmetric CH2 and CH3 (ASCC) region. TC1-4 and STC1-4 are four peaks in total carbohydrate (TC) and structural carbohydrate (STC) regions, respectively; CEC, cellulosic compounds region; SyCH2, symmetric CH2; SyCH3, symmetric CH3; AsCH2, asymmetric CH2; AsCH3, asymmetric CH3. W1a-1.csv, t2a-1.csv, h1a-1.csv are spectral examples of WT, TT8i and HB12i alfalfa. W1a-1-derivative.csv is the second derivative of w1a spectrum, showing as an example.

Prior to the FTIR spectra collection, freeze-dried alfalfa plants of different genotype were ground through a 0.02 mm sieve (Retsch ZM 200,Retsch Inc., Newtown, PA, USA). Afterwards, the background spectrum was measured with JASCO FT/IR-4200 with ATR (JASCO Corp., Tokyo, Japan) with 256 scans to minimize the CO_2_ noise. Then, the finely ground samples were directly placed on the crystal plate of JASCO FT/IR-4200 with ATR for spectra collection. Spectra were obtained at mid-IR region (ca. 4000–700 cm^−1^) at a resolution of 4 cm^−1^ with 128 scans (SpectraManager II software, JASCO Corp., Tokyo, Japan). Five subsamples of each sample were measured, generating five spectra for each sample. Background corrections were performed again after every five measurements to minimize the background noise. Spectra collection was conducted at the University of Saskatchewan, Canada. Figure 5 shows the example spectra of transgenic and WT alfalfa.

### 3.3. Univariate Analysis

**Figure 6:**
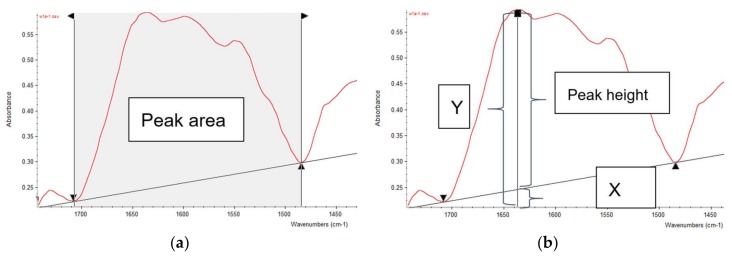
Illustrations of peak area and peak height measurements. (**a**) Peak area measurement. Corrected peak area equals the total area below the red spectrum minus the area below the baseline (straight black line); (**b**) Peak height measurement. Corrected peak height equals the total height (Y) minus the height below the baseline (X).

FTIR spectra were preprocessed by using OMNIC 7.3 software (Spectra Tech, Madison, WI, USA) before the measurements of peak heights and areas. First, each IR spectrum was normalized and then its second derivative was generated and auto-smoothed. The normalized spectra and smoothed second derivatives were then saved as “csv” files. Afterwards, all five spectra of each sample along with its second derivatives were read in excel and processed by using Excel^®^ macro for peak and area measurements. The Excel^®^ macro consisting of two Modules: 1. input all five csv-form spectra and five csv-form second derivatives into sheet1; 2. Automatically calculate peak heights and areas for each spectrum of five subsamples, then output the results into sheet2 (see Sup-1, macro template). Peak heights and areas were calculated with baseline correction (Figure 6). Peak heights equaled to total peak heights subtract the baseline heights at the peak wavenumber. Peak areas were calculated as total peak areas subtract the areas below the baseline. Total peak areas were determined as the cumulative area between every two adjacent wavenumbers under the spectrum, which were calculated as a trapezoidal shape. Wavenumbers of peaks and baseline points were determined according to the experiential wavenumbers [15,25,29]. 

Peaks and areas were measured in three regions: carbohydrate (CHO, ca. 1484–941 cm^−1^), Amide (ca. 1710–1484 cm^−1^) and lipid-related region (ca. 1781–1710 cm^−1^ and 3000–2761 cm^−1^). Within CHO region, wavenumbers were further divided into total carbohydrate (TC, ca. 1178–941 cm^−1^), structural carbohydrate (STC, ca. 1484–1178 cm^−1^), and cellulosic compounds (CEC, ca. 1283–1178 cm^−1^) regions. The TC region contains four major peaks at ca. 1149 (TC4), 1104 (TC3), 1074 (TC2), and 1026 (TC1) cm^−1^; STC also contains four major peaks at ca. 1453 (STC4), 1397 (STC3), 1370 (STC2), and 1317 (STC1) cm^−1^; CEC centers at ca. 1237 cm^−1^. Areas of each sub-carbohydrate regions (TCA, STCA, and CECA) were measured according to their baselines.

In amide region, unlike cereal spectra [17], sub-regions of amide I and amide II of alfalfa were overlapped in the current study (Figure 5a and Figure 6a). Therefore, a common baseline of 1710–1484 cm^−1^ was used to determine peak heights in amide region. For the heights of protein secondary structures, the second derivatives were used in assisting the determination. Amide I, amide II, α-helix and β-sheet peak at ca. 1649, 1540, 1653 and 1629 cm^−1^, respectively. Although no subdivision of amide region was performed, total amide area (AA) was divided into two subareas, amide I area (AIA) and amide II area (AIIA), by ca. 1575 cm^−1^. This was confirmed by a common peak in second derivatives of all IR spectra, and it also is the normal split point of amide I and II in cereal samples [17]. Moreover, ratios of some variables were calculated in amide region, including α-helix/β-sheet, amide I/amide II, AIA/AIIA, AIA/AA.

The lipid-related region contains two parts, carbonyl C=O region (CCO, ca. 1781–1710 cm^−1^) and (a)symmetric CH2 and CH3 region (ASCC, ca. 3000–2761 cm^−1^). The CCO centers at ca. 1733 cm^−1^, while ASCC features four major peaks at ca. 2955 cm^−1^ (asymmetric CH3, AsCH3), 2920 cm^−1^ (asymmetric CH2, AsCH2), 2872 cm^−1^ (symmetric CH3, SyCH3), and 2850 cm^−1^ (symmetric CH2, SyCH2). Areas of carbonyl C=O region (CCOA) and (a)symmetric CH2/CH3 region (ASCCA) were also measured according to their baselines.

### 3.4. Multivariate Analysis

Hierarchical cluster analysis (HCA) and principle component analysis (PCA) were performed on each region of IR spectra. In addition to all regions (CHO, TC, STC, CEC, amide, lipid) described above in univariate analysis, the whole spectrum region (ca. 4000–700 cm^−1^) and fingerprint region (ca. 1800–800 cm^−1^) were also analyzed. Both HCA and PCA were performed in R 3.4.2 software [32] with STATS package within Rsutdio^®^(RStudio Team, Boston, MA, USA) environment. Initially, all five spectra (csv files) of each sample were input into R software, and then spectra of all samples were integrated into one R object (one dataset). Afterwards, eight sub-datasets (four carbohydrate regions, one amide region, two lipid-related regions and one fingerprint region) were created from the whole dataset according to wavenumber range described previously. The HCA and PCA were then performed on all eight sub-regions, as well as on the whole region. For HCA, the mean spectra of each sample were calculated with aggregate() function in order to clarify the HCA cluster dendrogram. After that, dist() function was used for calculating the sample distance with Euclidean method. Function hclust() was then used for HCA clustering with Ward.D method by using squared Euclidean distance. The dendrograms of HCA were plotted with plot() function. All R functions mentioned above are from STATS package. For PCA, prcomp() function in STATS package was used with both *center* and *scale* options setting as true. Then, PCA plots were generated with ggbiplot() function with *ellipse* and *circle* options setting as true, and *var.axes* option setting as false, and options of o*bs.scale* and *var.scale* were set to 1. Function ggbiplot() was from GGBIPLOT package [33].

### 3.5. Statistical Analysis

Procedure MIXED of SAS 9.4 software (SAS Institute, Inc., Cary, NC, USA) was used to analyze univariate variables in IR spectra. The model used was $Y_{ijk}=\mu_{i}+\;geno_{i}+\;sub{\left(geno\right)}_{ij}\;+\varepsilon_{ijk}$, where *Y_ijk_* is the independent variable; *µ_i_* is the mean of all samples; *geno_i_* is the fixed genotype effect; *sub(geno)_ij_* is the random effect; *ε_ijk_* was the random error. Prior to variance analysis, a SAS macro with the same model was used to remove all outliers with a criterion of Studentized Residual greater than 2.5. Contrast statement was used to compare WT with transgenic alfalfa. The Tukey-Kramer method was used for multi-comparison between genotypes. Proc UNIVARIATE with norm and plot options was used to test the normality of the residue of each variable. Significance level was set as *p* < 0.05.

## 4. Conclusions

In conclusion, genetic transformation of alfalfa with TT8 and HB12 RNAi affected molecular spectral structures. Silencing of TT8 and HB12 affected both amide and carbohydrate intrinsic molecular structures in alfalfa, and such structural changes could be detected by ATR-FTIR spectroscopy. Both HCA and PCA multivariate analyses separated from transformed alfalfa in CHO region and ASCC lipid region, while all genotypes were successively separated in amide region.

## Figures and Tables

**Table 1:** Carbohydrate structural parameters of transgenic and wild type (WT) alfalfa.

Items	WT	Transgenic Alfalfa		SEM ^1^	*p*	Contrast ^2^ W vs. G
		HB12i	TT8i			
Total carbohydrate related spectral profiles ^3^						
TC1	0.646a	0.612b	0.606b	0.007	0.003	0.001
TC2	0.498b	0.55a	0.533a	0.0083	0.001	0.001
TC3	0.338b	0.381a	0.373a	0.0054	<0.001	<0.001
TC4	0.159	0.152	0.149	0.0028	0.088	0.035
TCA	76.091	78.571	77.296	0.9286	0.162	0.157
Cellulosic compounds related structural profiles ^4^						
CEC	0.078	0.072	0.077	0.0029	0.175	0.33
CECA	2.786	2.504	2.712	0.1841	0.467	0.48
Structural carbohydrate related structural profiles ^5^						
STC1	0.093b	0.106a	0.1ab	0.0026	0.007	0.014
STC2	0.151b	0.162a	0.152b	0.0028	0.006	0.122
STC3	0.164c	0.23a	0.187b	0.0048	<0.001	<0.001
STC4	0.131b	0.168a	0.143b	0.0043	<0.001	<0.001
STCA	28.035c	35.027a	30.747b	0.4989	<0.001	<0.001

Values with same letter in each row mean not significantly different at *p* > 0.05. ^1^ SEM, standard error of mean; ^2^ Contrast between wild type (WT) and transgenic alfalfa; ^3^ TC1–TC4, four major peaks at ca. 1026 (TC1) 1074 (TC2), 1104 (TC3) and 1149 (TC4) cm^−1^ in TC region, respectively; TCA, peak area of TC region; ^4^ CEC, cellulosic compounds (ca. 1237 cm^−1^); CECA, peak area of CEC region; ^5^ STC1–STC4, four major peaks at ca. 1317 (STC1), 1370 (STC2), 1397 (STC3) and 1453 (STC4) cm^−1^, respectively.

**Table 2:** Amide structural parameters in Amide region of transgenic and wild type (WT) alfalfa.

Items	WT	Transgenic Alfalfa		SEM ^1^	*P*	Contrast ^2^ W vs. G
		HB12i	TT8i			
Amide heights and spectral ratio profiles						
Amide I	0.356	0.341	0.348	0.0098	0.508	0.379
Amide II	0.272	0.269	0.241	0.0085	0.042	0.15
Amide I/Amide II	1.311b	1.258b	1.436a	0.0216	<0.001	0.232
Secondary structures heights and ratio profiles						
α-helix	0.346	0.333	0.333	0.0083	0.481	0.24
β-sheet	0.335b	0.386a	0.369a	0.0071	<0.001	<0.001
α-helix/β-sheet	1.041a	0.863c	0.907b	0.0106	<0.001	<0.001
Amide area and ratio profiles						
Amide area (AA)	49.183b	54.941a	50.052b	1.078	0.001	0.035
Amide I area (AIA)	33.168b	37.906a	35.545ab	0.7236	<0.001	0.002
Amide II area (AIIA)	16.016ab	16.991a	14.659b	0.4144	0.001	0.735
AIA/AIIA	2.072c	2.237b	2.42a	0.0351	<0.001	<0.001
AIA/AA	0.674c	0.691b	0.707a	0.0032	<0.001	<0.001

Values with same letter in each row mean not significantly different at *p* > 0.05. ^1^ SEM, standard error of mean; ^2^ Contrast between wild type (WT) and transgenic alfalfa.

**Table 3:** Lipid-related structural parameters of transgenic and wild type (WT) alfalfa.

Items	WT	Transgenic Alfalfa		SEM ^1^	*P*	Contrast ^2^ W vs. G
		HB12i	TT8i			
Carbonyl C=O ester height and area profiles ^3^						
CCO	0.063	0.059	0.056	0.0024	0.232	0.109
CCOA	1.827	1.69	1.657	0.0915	0.471	0.228
Symmetric and asymmetric of CH2 and CH3 profiles ^4^						
SyCH2	0.101b	0.133a	0.116b	0.0051	0.001	0.004
SyCH3	0.059	0.062	0.06	0.0014	0.189	0.214
AsCH2	0.184b	0.237a	0.206b	0.0084	0.001	0.004
AsCH3	0.061	0.066	0.062	0.0016	0.063	0.214
ASCCA	11.719b	13.387a	12.387ab	0.3509	0.007	0.024

Values with same letter in each row mean not significantly different at *p* > 0.05. ^1^ SEM, standard error of mean; ^2^ Contrast between wild type (WT) and transgenic alfalfa; ^3^ CCO, carbonyl C=O (centers at ca. 1733 cm^−1^); CCOA, peak area of CCO region (baseline ca. 1781–1710 cm^−1^); ^4^ SyCH2, symmetric CH2 (ca. 2850 cm^−1^); SyCH3, symmetric CH3 (ca. 2872 cm^−1^); AsCH2, asymmetric CH2 (ca. 2920 cm^−1^); AsCH3, asymmetric CH3 (ca. 2955 cm^−1^); ASCCA, peak area of asymmetric and symmetric CH2 and CH3 (baseline ca. 3000–2761 cm^−1^).

## Supplementary Materials

Supplementary materials can be found at http://www.mdpi.com/1422-0067/19/4/1046/s1. Sup-1: macro template; Sup-2: Small population size results.

## Abbreviations

TT8i	*TT8* silenced alfalfa
HB12i	*HB12* silenced alfalfa
WT	Wild type control alfalfa
TT8i	*Transparent Testa 8*-silenced alfalfa
HB12i	*Homeobox 12*-silenced alfalfa
TC	Total carbohydrate
TCA	TC peak area
STC	Structural carbohydrate
STCA	STC peak area
CP	Crude protein
SEM	Standard error of mean
CEC	Cellulosic compounds
CECA	CEC peak area
CHO	Total carbohydrate
CCO	Carbonyl C=O
CCOA	CCO peak area
AA	Whole amide area
AIA	Amide I peak area
AIIA	Amide II peak area
SyCH2	Symmetric CH2
AsCH2	Asymmetric CH2
SyCH3	Symmetric CH3
AsCH3	Asymmetric CH3
ASCC	Asymmetric and symmetric CH2 and CH3
ASCCA	ASCC peak area
HCA	Hierarchical cluster analysis
PCA	Principle component analysis
