# Supplementary material for: Molecular Structural Changes in Alfalfa Detected by ATR-FTIR Spectroscopy in Response to Silencing of TT8 and HB12 Genes
Source: Int J Mol Sci. 2018 Mar 31;19(4):1046. doi: 10.3390/ijms19041046 (PMC5979274; doi:10.3390/ijms19041046)
Supplement: Supplementary file 1 [file ijms-19-01046-s001.zip › Supplementary materials/Sup-1.1 The explanation of macro template.docx]

**User’s Guide of “Macro Template”**

Yaogeng Lei

Department of Animal and Poultry Science, University of Saskatchewan

**Use Procedure**

1. In general, original spectra were stored in one folder (“Spectra Source”) with all five spectra of each sample storing in an individual subfolder. The subfolders of each sample were named with its sample name (i.e. W1 for one replicate of WT).
2. To use the template, user opens the Excel template, then clicks the command button (“Auto Input & Cal”) in “Sheet2”. Then, in the pop-up window, selects the folder where the original spectra are stored (the “Spectra Source” folder in this case). After that, then select the folder where the results files will be stored (“Spectra Results” folder in this case).
3. Then, the results of each sample (five replicates) will output to “Sheet2” after spectral calculation. The results include peak heights, peak areas and peak regions (where the peak centers or where the baseline starts or ends). After calculation, the excel file will be saved in the result folder (“Spectra Results”). There will be one Excel file for each sample with the same name as the individual source folder (i.e. W1.xlsm for W1 subfolder).

**How the template works**

1. When the macro runs, it will initially input all five spectra and derivatives of each sample into “Sheet1”.
2. Then, the macro will use the experiential data in “Sheet3” to find the peak point and baseline of each peak.
3. Then, the macro writes spectral heights, areas, and wavenumbers of peaks and baselines to “Sheet2”.

**Worksheet Info**

1. **“Sheet1”** Stores spectra and secondary derivatives.
2. **“Sheet2”** Stores output of peak heights, area, and wavenumbers of peaks and baselines.
3. “**Sheet3”**
   1. Column “A” in “Sheet3” show the path where the spectra and its corresponding secondary derivatives are found.
   2. Column “C” to “E” stores the experiential wavenumbers of peaks and baseline starting and ending point.
   3. Column “F” and “G” are the up and down boundary (rows) of each item.
   4. Column “H” and “I” are the up and down boundary (wavenumbers) of each item.
