# Supplementary material for: Molecular Structural Changes in Alfalfa Detected by ATR-FTIR Spectroscopy in Response to Silencing of TT8 and HB12 Genes
Source: Int J Mol Sci. 2018 Mar 31;19(4):1046. doi: 10.3390/ijms19041046 (PMC5979274; doi:10.3390/ijms19041046)
Supplement: Supplementary file 1 [file ijms-19-01046-s001.zip › Supplementary materials/Sup-2 Small population size results.docx]

**Table 1. FTIR Carbohydrate Profiles of Transformed and WT alfalfa when only two population from each genotype were selected^*^.**

| **Items** | **WT** | **Transformed Alfalfa** | | **SEM^1^** | ***P*** | **Contrast^2^** |
| --- | --- | --- | --- | --- | --- | --- |
|  |  | **HB12** | **TT8** |  |  | **W vs G** |
| **Total carbohydrate profiles** | | | | | | |
| TC1 | 0.643 | 0.606 | 0.611 | 0.0125 | 0.22 | 0.109 |
| TC2 | 0.503 | 0.538 | 0.545 | 0.0136 | 0.209 | 0.104 |
| TC3 | 0.355 | 0.378 | 0.387 | 0.0097 | 0.204 | 0.106 |
| TC4 | 0.166 | 0.143 | 0.157 | 0.0057 | 0.134 | 0.099 |
| TCA | 77.937 | 77.934 | 80.384 | 1.6323 | 0.544 | 0.584 |
| **Cellulosic compounds profiles** | | | | | | |
| CEC | 0.1 | 0.089 | 0.094 | 0.002 | 0.084 | 0.048 |
| CECA | 4.93a | 4.505b | 4.719ab | 0.0701 | 0.05 | 0.032 |
| **Structural carbohydrate profiles** | | | | | | |
| STC1 | 0.106 | 0.114 | 0.108 | 0.0022 | 0.15 | 0.174 |
| STC2 | 0.161 | 0.163 | 0.156 | 0.0059 | 0.75 | 0.823 |
| STC3 | 0.175 | 0.219 | 0.197 | 0.0129 | 0.198 | 0.129 |
| STC4 | 0.132 | 0.141 | 0.139 | 0.0113 | 0.838 | 0.592 |
| STCA | 31.669 | 36.409 | 34.095 | 1.3903 | 0.198 | 0.126 |
| **Amide profiles** |  |  |  |  |  |  |
| AmideII | 0.27 | 0.247 | 0.24 | 0.0241 | 0.677 | 0.425 |
| Beta-sheet | 0.35 | 0.364 | 0.365 | 0.0153 | 0.779 | 0.515 |
| AmideI | 0.36 | 0.314 | 0.334 | 0.0146 | 0.232 | 0.139 |
| Alpha-helix | 0.349 | 0.309 | 0.317 | 0.0208 | 0.454 | 0.251 |
| Amide I/Amide II | 1.333 | 1.277 | 1.401 | 0.0783 | 0.593 | 0.953 |
| Helix/sheet | 0.998a | 0.861b | 0.867ab | 0.0232 | 0.041 | 0.018 |
| Amide Area (AA) | 49.62 | 51.913 | 49.973 | 2.9926 | 0.851 | 0.742 |
| Amide I Area (AIA) | 23.491 | 24.468 | 22.544 | 1.9557 | 0.799 | 0.996 |
| Amide II Area (AIIA) | 26.128 | 27.142 | 27.429 | 1.0835 | 0.702 | 0.447 |
| AIA/AIIA | 0.9 | 0.914 | 0.818 | 0.0386 | 0.307 | 0.526 |
| AIA/AA | 0.473 | 0.477 | 0.449 | 0.0115 | 0.315 | 0.532 |
| **Lipid-related profiles** |  |  |  |  |  |  |
| CCO | 0.064 | 0.053 | 0.06 | 0.0054 | 0.413 | 0.302 |
| SyCH2 | 0.099 | 0.117 | 0.118 | 0.0086 | 0.34 | 0.174 |
| SyCH3 | 0.06 | 0.058 | 0.061 | 0.0026 | 0.815 | 0.85 |
| AsCH2 | 0.182 | 0.215 | 0.21 | 0.0139 | 0.337 | 0.174 |
| AsCH3 | 0.06 | 0.058 | 0.058 | 0.0038 | 0.874 | 0.638 |
| CCOA | 1.852 | 1.615 | 1.837 | 0.1463 | 0.521 | 0.533 |
| ASCCA | 11.667 | 12.537 | 12.604 | 0.7383 | 0.648 | 0.391 |

Note: Two populations of each alfalfa genotype were selected in this analysis. WT, W2 and W3; TT8i, T2 and T3; HB12i, H2 and H3;

^1^ SEM, standard error of mean.  ^2^ Contrast between WT and transgenic alfalfa; Values with same letter in each row mean not significantly different at P>0.05.

TC1-TC4, four major peaks at ca. 1026 (TC1) 1074 (TC2), 1104 (TC3) and 1149 (TC4) cm-1 in TC region, respectively; TCA, peak area of TC region.

CEC, cellulosic compounds (ca. 1237 cm-1); CECA, peak area of CEC region.

STC1-STC4, four major peaks at ca. 1317 (STC1), 1370 (STC2), 1397 (STC3) and 1453 (STC4) cm-1, respectively;

CCO, carbonyl C=O (centers at ca. 1733 cm^-1^); CCOA, peak area of CCO region (baseline ca. 1781-1710 cm^-1^).

SyCH2, symmetric CH2 (ca. 2850 cm^-1^); SyCH3, symmetric CH3 (ca. 2872 cm^-1^); AsCH2, asymmetric CH2 (ca. 2920 cm^-1‑^); AsCH3, asymmetric CH3 (ca. 2955 cm^-1^); ASCCA, peak area of asymmetric and symmetric CH2 and CH3 (baseline ca. 3000-2761 cm^-1^).
